# Supplementary material for: End Sequence Analysis Toolkit (ESAT) expands the extractable information from single-cell RNA-seq data
Source: Genome Res. 2016 Oct;26(10):1397–410. doi: 10.1101/gr.207902.116 (PMC5052061; doi:10.1101/gr.207902.116)
Supplement: Supplemental Material [file supp_gr.207902.116_Supp_Materials.tar.gz › ESAT-esat_v0.1/doc/html/classbroad_1_1projection_1_1gsa_1_1_gene_set_enrichment-members.html]

ESAT: Member List


ESAT


- Main Page
- Related Pages
- Packages
- Classes
- Files

- Class List
- Class Index
- Class Hierarchy
- Class Members

All Classes Namespaces Files Functions Variables Enumerator Pages

- broad
- projection
- gsa
- GeneSetEnrichment

broad.projection.gsa.GeneSetEnrichment Member List

This is the complete list of members for broad.projection.gsa.GeneSetEnrichment, including all inherited members.

|  |  |  |
| --- | --- | --- |
| GeneSetEnrichment(MatrixWithHeaders data, Collection< String > group1, Collection< String > group2, Map< String, Collection< String >> geneSets, boolean normalizeScores, boolean permutePhenotype) | broad.projection.gsa.GeneSetEnrichment |  |
| GeneSetEnrichment(MatrixWithHeaders data, Collection< String > group1, Collection< String > group2, Map< String, Collection< String >> geneSets) | broad.projection.gsa.GeneSetEnrichment |  |
| GeneSetEnrichment(MatrixWithHeaders patternData, MatrixWithHeaders geneData, Map< String, Collection< String >> geneSets, boolean normalizeScores, boolean permutePhenotype) | broad.projection.gsa.GeneSetEnrichment |  |
| getKSEnrichments() | broad.projection.gsa.GeneSetEnrichment |  |
| getKSFDR() | broad.projection.gsa.GeneSetEnrichment |  |
| getMaxMeanEnrichments() | broad.projection.gsa.GeneSetEnrichment |  |
| getMaxMeanFDR() | broad.projection.gsa.GeneSetEnrichment |  |
| getNormalizedKSEnrichments() | broad.projection.gsa.GeneSetEnrichment |  |
| getNormalizedMaxMeanEnrichments() | broad.projection.gsa.GeneSetEnrichment |  |
| getRankedList() | broad.projection.gsa.GeneSetEnrichment |  |


---

Generated on Fri Aug 15 2014 15:11:05 for ESAT by  

 1.8.7
